# Biomechanical Assessment of a Patient-Specific Knee Implant Design Using Finite Element Method

**DOI:** 10.1155/2014/353690

**Published:** 2014-06-30

**Authors:** Qiguo Rong, Jianfeng Bai, Yongling Huang, Jianhao Lin

**Affiliations:** ^1^College of Engineering, Peking University, Beijing 100871, China; ^2^Baimtec Material Co., LTD., Beijing 100095, China; ^3^Peking University People's Hospital, Beijing 100044, China

## Abstract

Rheumatoid arthritis is the leading cause of disability in young adults. Total knee arthroplasty has been successfully used to restore the joint function. Due to small bone size, osteoporosis, and severe soft tissue disease, standard knee implant sometimes cannot be directly applied clinically and patient-specific designs may be a more rational choice. The purpose of this study was to evaluate the biomechanical behavior of a patient-specific knee implant. A three-dimensional finite element of total knee arthroplasty was developed. The mechanical strength and the wear damage of the articular surfaces were analyzed. The results show that there exist high risks of component fracture and wear damage; the proposed implant design should be abandoned. The presurgery analysis is helpful in avoiding the potential failure.

## 1. Introduction

Total knee arthroplasty (TKA) is now a routine treatment procedure for patients with knee osteoarthritis, rheumatoid arthritis, osteonecrosis, and other types of inflammatory arthritis. Continual advancements in the knowledge of knee mechanics have led to design modifications that broaden range of indications. Many new kinds of knee implant designs have been developed in the last few decades. More and more patients are receiving the benefits of total knee arthroplasty. Despite all these progresses, clinically applied standard designs meet sometimes difficulties in special cases, for example, by patients with severe joint deformities. Therefore, patient-specific designs appear to be a reasonable solution.

The design of knee implant must follow the biomechanical principle, avoiding loosening, abrasion, or fracture. A patient-specific implant cannot undergo all design and test processes demanded by a standard one because of high costs. Therefore, the presurgery evaluation of a patient-specific knee implant is usually done by CAD software and finite element method.

Juvenile rheumatoid arthritis is a type of arthritis that often affects the function of knee joint. Over the past 30 years, total knee arthroplasty has been applied to relieve pain and restore function of knee. Some patients need custom-made components due to small bone size, osteoporosis, and severe soft-tissue disease [1]. The long-term results of total knee arthroplasty in patients with juvenile rheumatoid arthritis have been shown to be successful in relief of pain and improvement in function [2–4].

The purpose of this study was to evaluate the mechanical behavior of a hinged knee implant designed for a patient with rheumatoid arthritis. The finite element model of the knee implant was established and the stress characteristics of all implant parts under functional loading were analyzed.

## 2. Methods

A 20-year-old female patient suffers from severe rheumatoid arthritis. Her hip and knee joints lost the movement ability and became rigid. After hip replacement surgery, she needs now to undergo the bilateral knee replacement surgery to reestablish the joint function. The question is what kind of knee implant can fit her special joint conditions and guarantee the long-term success.

**Figure 1:**
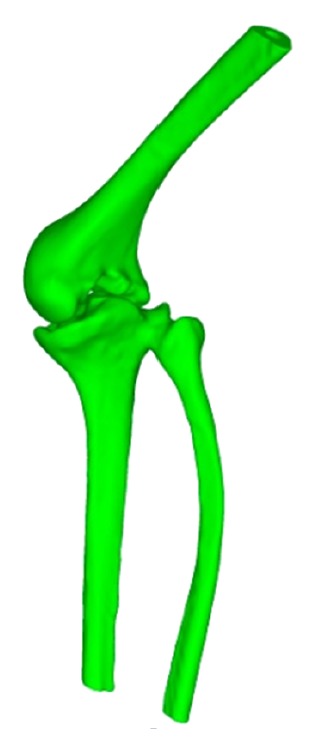
The rigid bonding of femur and tibia of the patient.

Long time immobilization in bed caused the atrophy of bone and muscle.  Figure 1 shows the rigid bonding of the knee joint. The distal part of the femur and the pulp cavity are much smaller than normal ones. The femur of the patient has a diameter of about 16 centimeters for the shaft and a diameter of about 9 centimeters for the pulp cavity. The mechanical tibiofemoral angle is also larger than normal alignment. The shape and size of the die cavity are usually determined by the measure of normal knee joint structures. Even the smallest standard knee implant cannot be directly applied to this patient. The key factors in total knee arthroplasty are the joint surface matching of femoral and tibial components on the one hand and the installation firmness on the other hand. Therefore, the necessary amount of bone should be kept after cut of the distal femur and proximal tibia. The conventional prosthesis cannot fulfil the special conditions of this patient. An adaptation of the implant design should be done according to the patient conditions.

**Figure 2:**
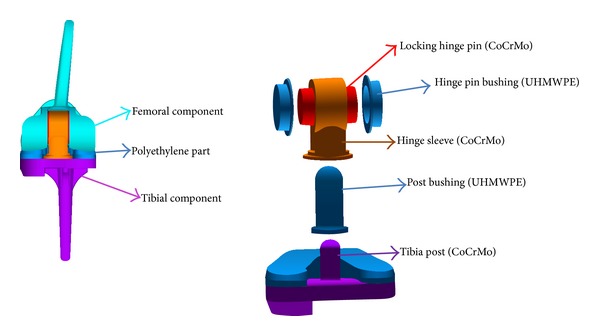
The patient-specific design of a hinged knee implant.

In consideration of the limited muscle function, a traditional hinged implant was first chosen as the candidate. The modified design of the knee implant was shown in Figure 2. The diameter of the femur part is from 7 to 10 millimeter. The length of the femur stem is about 6 centimeter. The length of the tibia stem is about 7 centimeter. The diameter of the tibia part is from 7 to 13 millimeter.

The knee implant is a complex kinematic mechanism, which includes multiple contact pairs: (1) the condyle bearing surfaces (femur part) and the abutting bearing surfaces (polyethylene part); (2) the tibia post and the post bushing; (3) the post bushing and hinge sleeve; (4) the post busing and the locking hinge pin; (5) the locking hinge pin and the hinge sleeve; and (6) the locking hinge pin and the hinge pin bushing. All these contact pairs will influence the force transmission and stress distribution at different degrees.

The condyle bearing surfaces maintain contact across the abutting bearing surface of the meniscal component throughout the full range of motion. This contact pair is also considered the most important for the long success of the implant, because the wear damage may cause the failure of the joint articulation. Another important contact pair is the tibial post bushing and the relevant parts, which should be carefully evaluated to avoid the structure fracture. Other contact pairs are less important because they do not bear functional loads.

**Figure 3:**
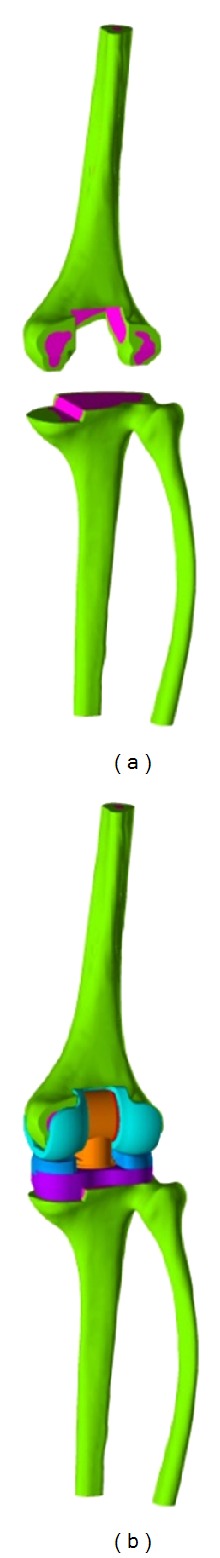
The finite element model of the total knee arthroplasty. (a) The cutting of bones and (b) restoration by the hinged knee implant.

A three-dimensional finite element model of the total knee replacement was developed in ANSYS. The femur and tibia were resurfaced to fit the implant parts and then were repositioned to mimic the surgical results.  Figure 3(a) shows the femur and tibia bones after resurfacing. The whole finite element model includes 252868 nodes and 175979 elements, as shown in Figure 3(b).

The femoral and tibial components are made of a cobalt-chromium-molybdenum (CoCrMo) alloy. Ultra-high-molecular-weight polyethylene (UHMWPE) is chosen for the polyethylene part.  Table 1 gives the material parameters for the implant parts and bone tissues.

**Figure 4:**
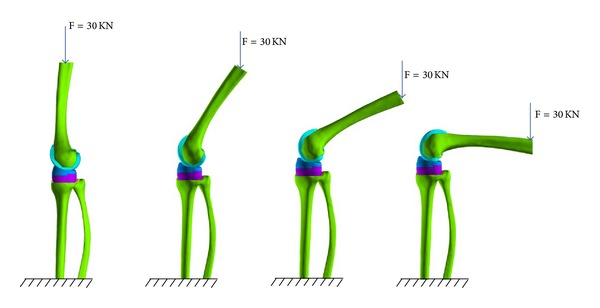
Loading conditions for flexion angle of 0°, 30°, 60°, and 90°. The base of the tibia was fixed and the vertical load was applied to the upper end of femur.

The fixed boundary condition was specified on the base of the tibia. The femoral component was constrained to keep contact with the articular surface. It can rotate within the range of flexion angle from −3° to 125°. The body weight of the patient is about 300 N. A vertical load of 300 N was applied to the femur end, which corresponded to the state of standing on one leg (Figure 4). The interface frictional characteristic between the polyethylene insert and femoral component was assumed to be frictionless. Eight simulations were performed for the flexion angle of −3°, 0°, 10°, 20°, 30°, 60°, 90°, and 125°.

## 3. Results

**Figure 5:**
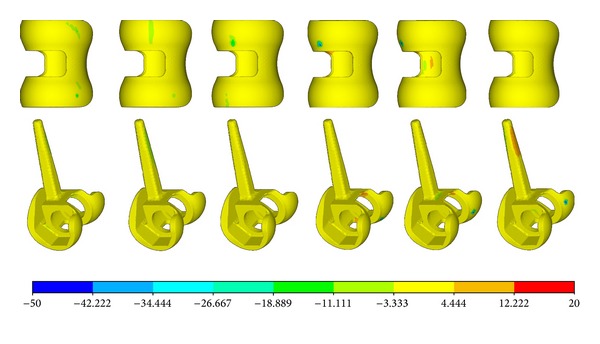
The *σ*_*zz*_ distribution of the femur part.

**Figure 6:**
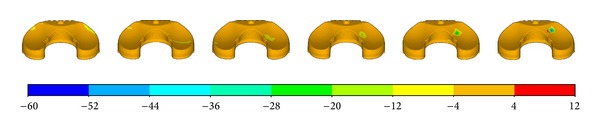
The *σ*_*zz*_ distribution of the plate.

The largest component of the stress tensor is usually the vertical one *σ*_*zz*_, so it was chosen as the criterion for biomechanical evaluations.  Figure 5 shows the *σ*_*zz*_ distribution of the femur component. The contact position located at the anterolateral part of the condyle bearing surfaces at −3° flexion and moved posteromedially with the increasing of the flexion angle. The relationship between contact position and flexion angle was a little different for the abutting bearing surface as shown in Figure 6. The contact position occurred at the anterior outer edge of the articular surface at flexion angle of −3°. It moved abruptly to the middle part of the articular area when it was without flexion. And then, the contact position moved anteromedially with the increasing of flexion angle. It can also be easily observed that the contact pressure is not symmetrical. The lateral side bore more loads than the medial sides.

**Figure 7:**
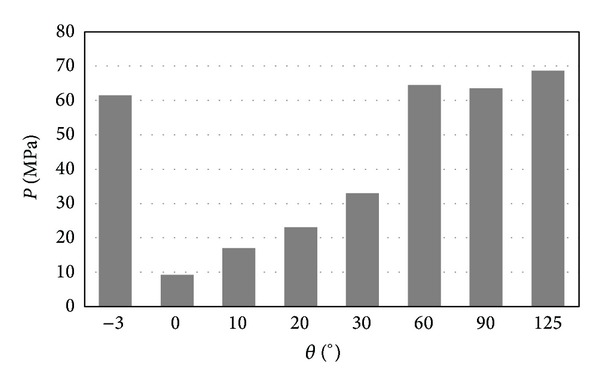
The relationship between the maximum contact pressure and the flexion angle.

**Figure 8:**
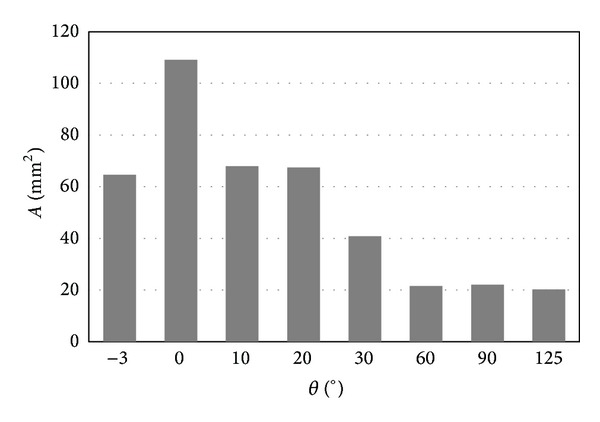
The relationship between the contact area and the flexion angle.

The relationship between pressure and flexion angel was more clearly demonstrated by Figure 7. It was easy to observe that the most critical condition was either the negative flexion or the large flexion. For the articular surfaces, the contact pressure was more than 60 MPa for flexion of −3° and flexion of more than 60°.  Figure 8 shows the relationship between contact area and flexion angel. It can be easily observed that the contact pressure is inversely proportional to the contact area. The smaller the contact area is, the higher the contact pressure is.

**Figure 9:**
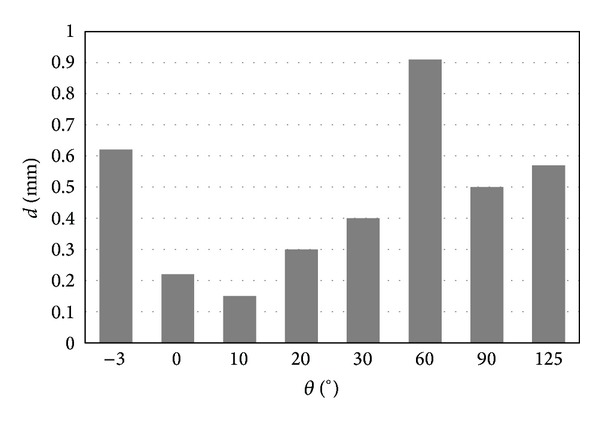
Sliding distance of the contact parts.

Figure 9 shows the maximum slip displacement of the femur part across the polyethylene part. The largest slipping occurred at the flexion of 60°, while the smallest slipping occurred at the flexion of 10°.

**Figure 10:**
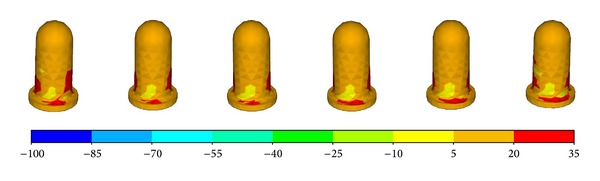
The *σ*_*zz*_ distribution of the post bushing.

**Figure 11:**
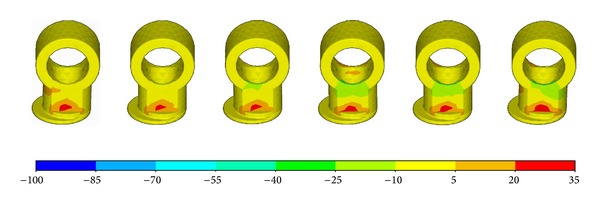
The *σ*_*zz*_ distribution of the hinge sleeve.

For post bushing and hinge sleeve, the maximum stress occurred at the parts with sharp edges, as shown in Figures 10 and 11.

**Figure 12:**
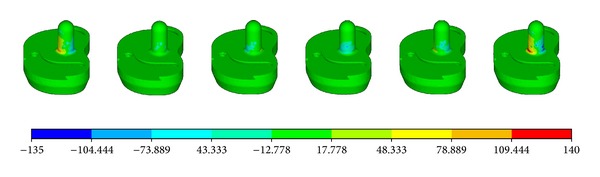
The *σ*_*zz*_ distribution of the tibia post.

**Figure 13:**
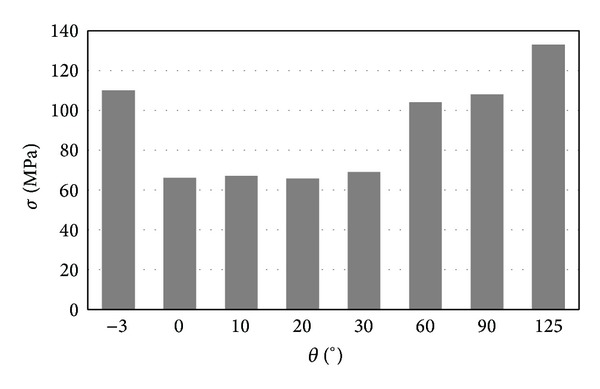
The relationship between the maximum *σ*_*zz*_ and the flexion angle.

The highest stress was observed at the tibia post, which was the main structure component to guarantee the stability of the knee implant.  Figure 12 shows that the stress concentration occurred mainly at the base of the post. The value of *σ*_*zz*_ can reach −135 MPa for pressure and 140 MPa for tension, as shown in Figure 13.

## 4. Discussion

From mechanical point of view, the failure of knee implant was caused mainly by two problems, namely, the wear of the polyethylene part and the strength of implant components.

The loading condition was simplified in this study for two reasons: (1) the purpose of this study was to evaluate the practicability of the implant design, not to simulate the functional activities of knee joint after TKA, and a roughly approximative load can satisfy the need; (2) muscles and ligaments play important roles in the knee statics and dynamics, but the long-term immobilization inevitably leads to degenerative changes of relevant muscles and ligaments; it is therefore not possible to estimate the realistic function of the muscles and ligaments of the patient in this study reliably.

The loads in the knee joint can vary dramatically during different activities. For a healthy subject, the tibiofemoral load is from 3 to 4 body weight (BW) for walking, from 4 to 6 BW for stair climbing, and up to 8 BW for walking downhill. The vertical load of 300 N used in this study is only about 1 BW. The patient in this study cannot walk dynamically like a healthy subject, so the load can be assumed approximately to be equivalent to the walking load.

The contact area of a normal human knee varies from 750 to 1150 mm^2^, and the contact stress was usually less than 5 MPa. The contact area of the most fixed knee implant is much smaller, only about 10 mm^2^~300 mm^2^. Contrarily, the contact stress increases to about 60 MPa. Since the maximal fatigue strength of modern polyethylene materials is less than 21 MPa. The ideal contact stress of the joint contact surfaces should be less than 10 MPa. The simulation showed that the contact pressure is within the safety range only for very small flexion angles. For negative flexion and deep flexion, the contact pressure is higher than 60 MPa, which will lead to wear of the polyethylene components in total knee arthroplasties, a potential long-term problem. van den Heever et al. analyzed the contact stresses in a patient-specific unicompartmental knee replacement [5]. The maximum contact stresses usually occurred at 45° flexion. But the stress level is much lower, about one third of this study.

In some studies, the wear depth at a surface point of the polyethylene components can be estimated by Archard's wear law [6, 7]:

(1) $$\begin{array}{c} H_{\text{wear}}=kpd, \end{array}$$

where *H*_wear_ is the wear depth, *k* is the wear factor, *p* is the contact pressure, and *d* is the sliding distance between the two components. The calculated wear factor *k* was about 1.03 × 10^−7^ mm^3^/Nm [6]. Due to the large sliding displacement, the most critical flexion angel is about 60°. In order to reduce the wear damage, the knee implant with mobile bearings may be another candidate for the patient. For some new knee implant with mobile bearings, the contact area can increase to 600~700 mm^2^ and keep the contact stress less than 5 MPa. But how to resolve the stability problem is also a difficult task.

Fixed uniaxial hinge prostheses can give stability to the diseased knee; however, excess stress applied to the bone and articular surfaces may lead to early osteolysis and loosening of the components. The fracture of the tibial metal post was also reported recently [8]. It is therefore necessary to examine the stress distribution in implant components more critically.

The simulations show that the most possible failure occurred at the tibia post. The contact situation between the tibia post and the post bushing is very complex. The tibia post bears very high bending moment. The strength of CoCrMo alloy is about 665 MPa and the yield strength is about 450 MPa. Though the maximum stress of 138 MPa is only about one-third of the yield strength, it is, nevertheless, still very dangerous, if the knee joint encounters a sudden loading from 7 to 8 BW, for example, in occasion of falling. Even for stair climbing, the bending stress of the tibia post may exceed the materials yield strength.

It is important to recognize that some limitations exist in this study. Several assumptions and simplifications were made in the computational model. Some failure types were not considered in this study, for example, abrasion from cement or bone. But the simulation results can already give the final conclusions.

## 5. Conclusions

The simulation results show that the patient-specific design of the hinged knee implant faces the risk of sudden break and the risk of long-term wear damage. It should therefore be abandoned and a new design should be looked for. Though the evaluation result is negative, it is valuable and important for patient-specific treatment.

## Figures and Tables

**Table 1:** Material properties applied to the FE model.

Material	Young's modulus (MPa)	Poisson's ratio
Cortical bone	13700	0.3
Cancellous bone	1370	0.25
Cement	18000	0.3
CoCrMo Alloy	220000	0.3
UHMWPE	1800	0.3
